# Supplementary material for: Multiband charge density wave exposed in a transition metal dichalcogenide
Source: Nat Commun. 2021 Oct 15;12:6037. doi: 10.1038/s41467-021-25780-4 (PMC8519912; doi:10.1038/s41467-021-25780-4)
Supplement: Supplementary file 1 — Supplementary Information [file 41467_2021_25780_MOESM1_ESM.pdf]

Supplementary Information for Multiband charge density wave exposed in a transition metal dichalcogenide

Árpád Pásztor,[1,*] Alessandro Scarfato,[1] Marcello Spera,[1] Felix Flicker,[2,3,4] Céline Barreteau,[1] Enrico Giannini,[1] Jasper van Wezel,[5] and Christoph Renner[1,†]

[1] *Department of Quantum Matter Physics, Université de Genève, 24 quai Ernest Ansermet, CH-1211 Geneva 4, Switzerland.*

[2] *Rudolph Peierls Centre for Theoretical Physics, University of Oxford, Department of Physics, Clarendon Laboratory, Parks Road, Oxford OX1 3PU, United Kingdom*

[3] *School of Physics and Astronomy, Cardiff University, Cardiff CF24 3AA, United Kingdom*

[4] *School of Mathematics, University of Bristol, Bristol BS8 1TW, United Kingdom*

[5] *Institute for Theoretical Physics Amsterdam and Delta Institute for Theoretical Physics, University of Amsterdam, Science Park 904, 1098 XH Amsterdam, The Netherlands*

CONTENTS

I. Fourier-filtering to separate the atomic lattice and CDW contributions to the STM topography. 3

II. Histograms of the dephasing parameter 3

III. Mapping the dephasing parameter to the phase of a unidirectional CDW 4

IV. Bias dependence of the phase away from defects 5

V. Bias dependence of the CDW phase in the vicinity of defects 6

VI. The CDW imaging amplitude 8

VII. Spatial and energy dependence of the LDOS in a 1D CDW 9

VIII. Calculation of the constant current topography 10

IX. Bias dependence of the CDW amplitude in the different models 12

X. Optimized parameters of the two-gap model 13

XI. Phase and amplitude in the three-gap model 14

References 15

* arpad.pasztor@unige.ch

† christoph.renner@unige.ch

I. FOURIER-FILTERING TO SEPARATE THE ATOMIC LATTICE AND CDW CONTRIBUTIONS TO THE STM TOPOGRAPHY.

We use Fourier filtering to separate the atomic lattice and CDW signals in topographic STM images. We start with the Fourier transform (FT) of the STM image, e.g. Fig. 2a of the main text. This yields a sharp and intense peak structure at the first order Bragg-peaks of the CDW and atomic modulations outlined in Suppl. Fig. 1a with red and blue circles, respectively. To obtain the image corresponding to the atomic (CDW) lattice, we mask the atomic (CDW) component by suppressing the values outside the blue (red) circles in the FT and we take the inverse transform of this modified map. These operation are always performed on the large scale STM images (Figs. 2a, b and c).

Suppl. Figs. 1b-d show magnified regions of the Fourier filtered STM images in Figs. 2a-c. They reveal the atomic lattice at the exact same locations as the CDW contributions shown in Figs. 2e, g and i, respectively. The atomic lattice contribution is the same in all three images, supporting the conclusion that the contrast changes in Figs. 2d, f and h are primarily due to changes in the CDW contrast with bias.

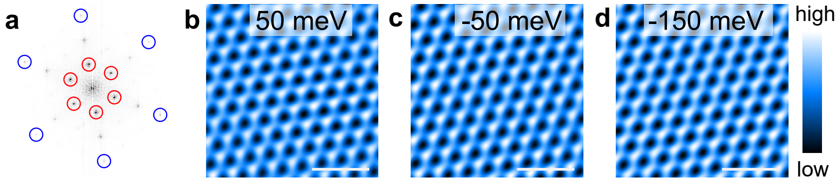

Suppl. Fig. 1. **Atomic lattice contribution to the STM topography.** **a** Fourier-transform of the STM image shown in Fig. 2a of the main text. The blue and red circles mark the peaks corresponding to the atomic and CDW components, respectively. Magnified Fourier filtered images of the atomic lattice at **b** $V_b = 50$ mV, **c** $V_b = -50$ mV and **d** $V_b = -150$ mV corresponding to the exact same region as shown in Figs. 2d-i of the main text. Scalebar: 1 nm.

II. HISTOGRAMS OF THE DEPHASING PARAMETER

Suppl. Figs. 2a, b, and c show the histograms of the dephasing parameter maps $\Theta(\mathbf{r})$ shown in Figs. 2j, k, and l in the main text, respectively. They all exhibit a well defined peak, which we fit with a Gaussian to determine the most frequent dephasing parameter

$\Theta_0(V_b)$ for each imaging bias.

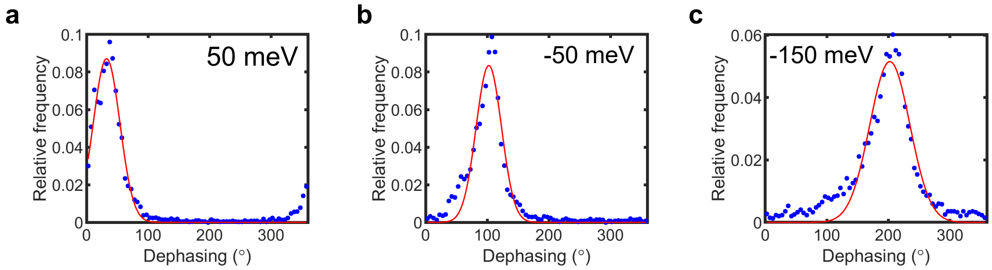

Suppl. Fig. 2. **Histograms of the dephasing parameter maps.** **a**, **b**, and **c** corresponds to the $\Theta(\mathbf{r})$ maps shown in Figs. 2j, k, and l in the main text, respectively. The solid red lines show the Gaussian fit of the dominant peak in each histogram. The peak position defines the representative dephasing parameter $\Theta_0(V_b)$ for each imaging bias V_b.

III. MAPPING THE DEPHASING PARAMETER TO THE PHASE OF A UNIDIRECTIONAL CDW

The appearance of the CDW pattern observed by STM can be reproduced by the sum of three plane waves [1]. The dephasing parameter $\Theta(\mathbf{r}) = \varphi_1(\mathbf{r}) + \varphi_2(\mathbf{r}) + \varphi_3(\mathbf{r}) \bmod 360°$, where $\varphi_i(\mathbf{r})$ is the phase of each plane wave, is a unique representation of a given CDW pattern. Any arbitrary combination of $\varphi_i(\mathbf{r})$, $i = (1, 2, 3)$, summing up to the same dephasing parameter, will describe this CDW pattern. In particular the combination where all three phases are equal. Based on the threefold symmetry of the system, there is no preferred direction and one may further assume that all three follow the same bias dependence.

These observations allow us to map $\Theta(\mathbf{r})$ to a one-dimensional problem we can more easily model. We start from $\Theta_0(V_b)$ (Suppl. Fig. 3a) obtained for each bias as shown in Suppl. Fig. 2. Next, we remove any phase jumps due to the 360° periodicity of $\Theta_0(V_b)$ (Suppl. Fig. 3b). Finally, we divide the resulting phase by three to obtain a representative one-dimensional (1D) phase $\varphi_0(V_b) = \Theta_0(V_b)/3$ (Suppl. Fig. 3c). Note that since the dephasing parameter $\Theta_0(V_b)$ is 360° periodic, we can freely add an integer times 120° to $\varphi_0(V_b)$ and still get the same dephasing parameter.

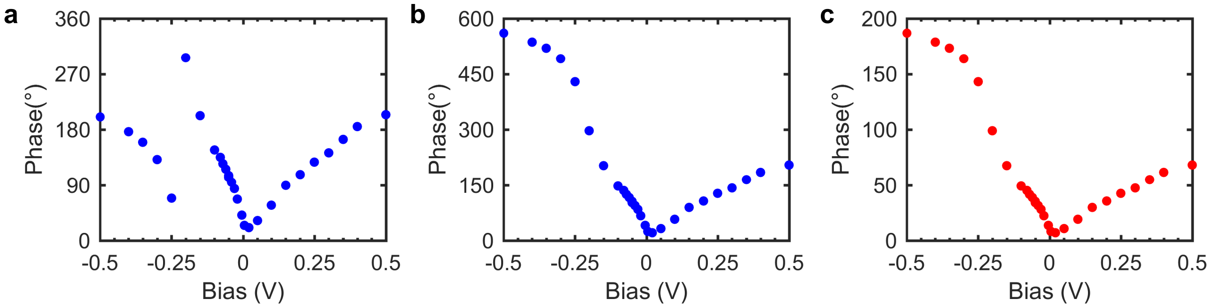

Suppl. Fig. 3. **Mapping the dephasing parameter to the phase of a unidirectional CDW.** **a** Dephasing parameter $\Theta_0(V_b)$ mod 360° as obtained from the local fitting of the real space charge modulation. The jump at -0.2 V is due to the 360° periodicity. **b** To remove the jump at -0.2 V, we add 360° to $\Theta_0(V_b)$ for V_b <-0.2 V (extended zone scheme). **c** $\varphi_0(V_b) = \Theta_0(V_b)/3$ mapping the extended zone dephasing parameter to the phase of a unidirectional CDW.

IV. BIAS DEPENDENCE OF THE PHASE AWAY FROM DEFECTS

Here we demonstrate that the scheme to extract the bias dependent phase from topographic STM images is robust. The analysis in the main text is based on the phase extracted from the entire field of view (FOV) of Figs. 2a, b and c. This FOV combines regions away from defects with different topographic patterns. Here we demonstrate that although each region may correspond to a slightly different phase value, the bias dependence of the phase remains the same.

In Suppl. Fig. 4a, we highlight eight regions from which we extract the CDW phase as a function of bias plotted in Suppl. Fig. 4b. The inset of panel b shows the same data offset for clarity. For the small regions (1-4), there are not enough points for a reasonable histogram. Therefore, we use a spatial average for the dephasing parameter in these regions. In the large regions, we extract the phase as described in the main text: we fit a Gaussian to the histogram of the dephasing parameter $\Theta(\mathbf{r})$ to determine the most frequent one $\Theta_0(V_b)$. We are aware that in general the most frequent value and the average may not coincide, but this was not a problem for the areas with homogeneous $\Theta(\mathbf{r})$ selected here.

Independently of the size or the precise location of the defect-free area from which we extract the phase, we always find the same bias dependence within a small spread. This is most remarkable when comparing regions that appear differently in the STM image,

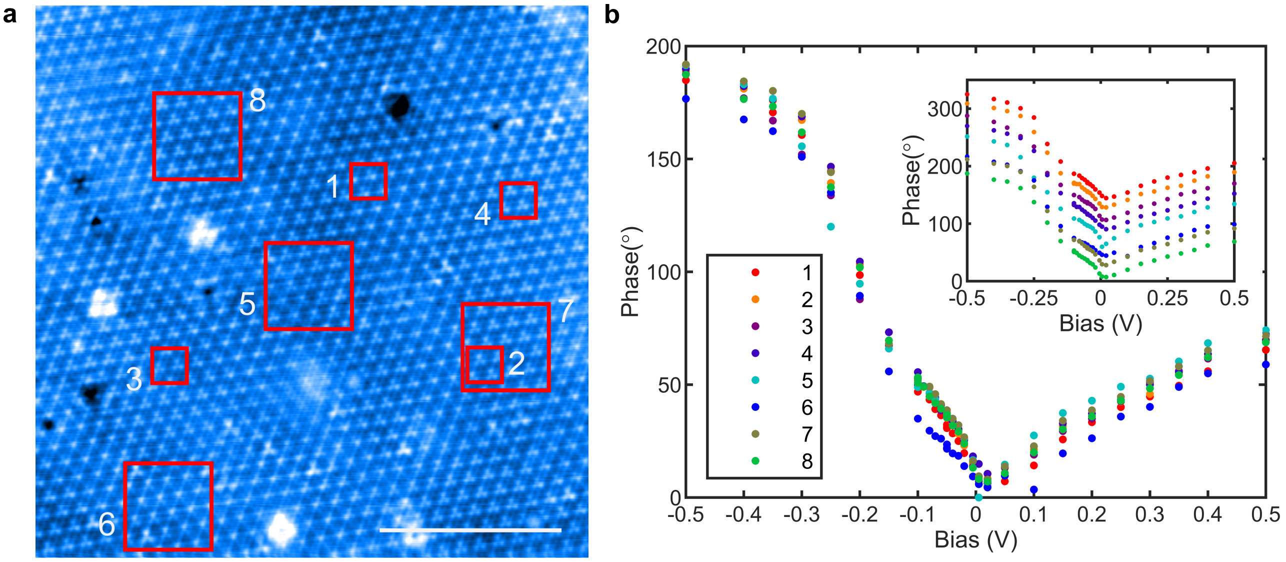

Suppl. Fig. 4. **Phase extracted away from defects.** **a** The same STM image as in Fig. 2a with numbered red squares marking the different areas where we extract the phase. Scalebar: 10 nm. **b** STM imaging bias dependence of the phase extracted at the marked regions. Inset: the same curves with a vertical offsets for clarity.

e.g regions 1 and 4 or regions 6 and 7. As the dephasing parameter only accounts for the appearance of the CDW pattern alone and not for its registry to the atomic lattice, the above observations imply that the CDW is composed of areas (connected by discommensuration with rapidly varying phase [1, 2]) where the CDW pattern is laterally shifted with respect to the atomic lattice. This is consistent with recent studies finding CDW domains with identical internal structures but with a distinct registry to the atomic lattice [3, 4].

V. BIAS DEPENDENCE OF THE CDW PHASE IN THE VICINITY OF DEFECTS

The impact of defects and impurities on the CDW is the focus of numerous studies, and is beyond the scope of the present investigation. Defects can act as strong pinning centers [5, 6], locking the local CDW to a particular phase or breaking it up into phase domains [7, 8]. In line with these studies, our analysis shows a very different behaviour of the CDW phase in the vicinity of defects, with a different and often weaker dependence on the imaging bias. This is illustrated in Suppl. Fig. 5 for a selection of defects. Similar looking defects in

the topography (Suppl. Fig. 5a) can drive a similar bias dependence of the CDW phase in their vicinity (defects 2 and 4), but also very different ones (e.g. defects 1 and 3, or 6 and 7). The latter may reflect de-pinning and re-pinning of the CDW at a given defect site in response to the repeated scanning of the same area at different biases.

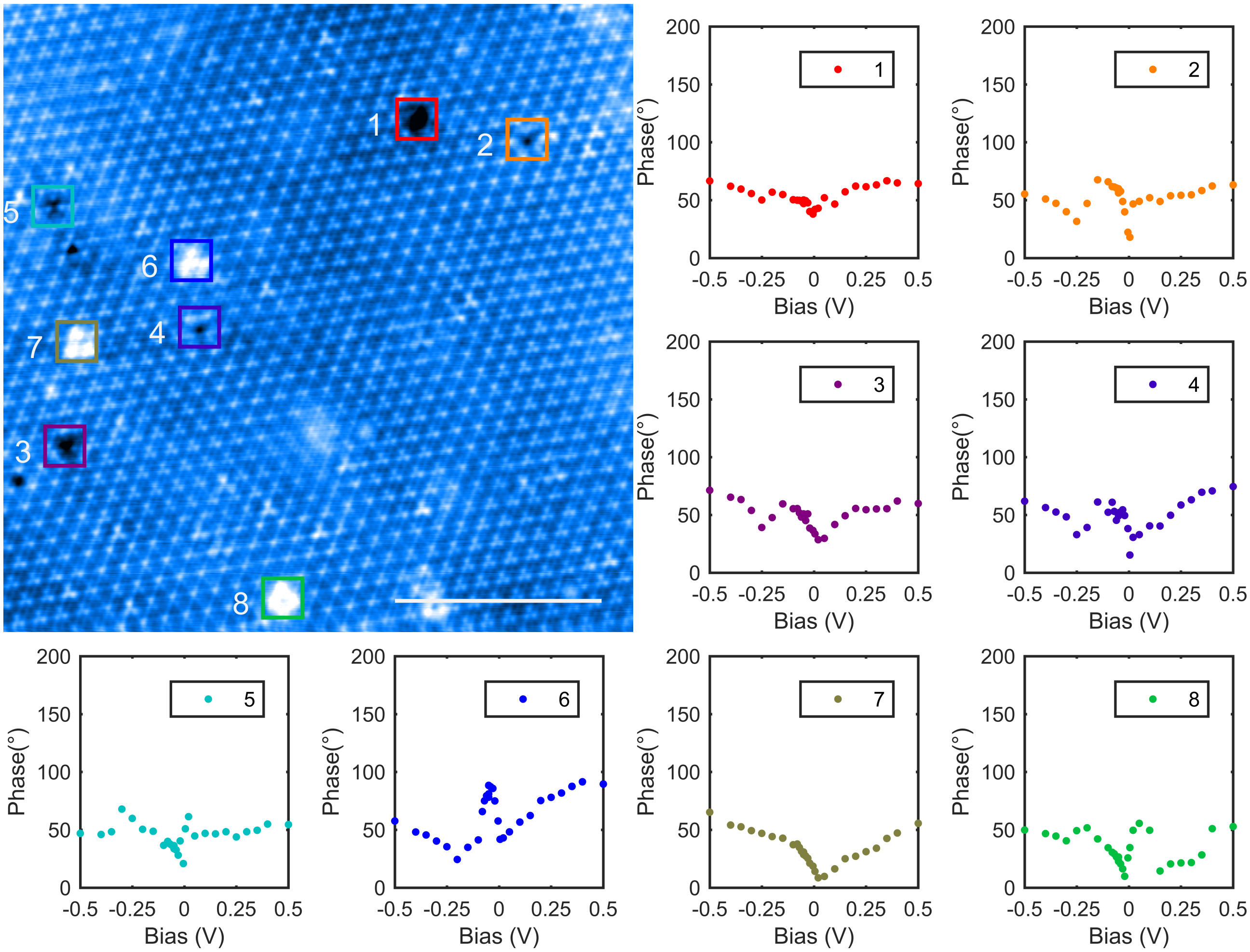

Suppl. Fig. 5. **Phase extracted around defects.** Main panel: the same STM image as in Fig. 2a with numbered coloured squares marking the different areas where we extract the phase. Scalebar: 10 nm. Small panels: the STM imaging bias dependence of the phase extracted at the marked regions.

Suppl. Fig. 5 graphically shows the importance of carefully considering defects in our analysis of the bias dependent CDW contrast. While the bias dependence varies a lot near different defects, it is consistently the same in the defect-free regions, independent of their location in the defect landscape (see Suppl. Fig. 4). This is a a very strong indication that

the defect-free regions we consider reveal intrinsic properties of the CDW modulation in $NbSe_2$. These regions are exempt of any local spectroscopic or topographic features at some specific bias that would be expected in presence of some hidden or subsurface defects. Moreover, the CDW Fourier peaks are sharp and well defined, unlike the characteristic signatures expected if defects were affecting the clean regions (see e.g. [9] and [10]). Finally, if strain due to the proximity to the visible defects or due to some hidden subsurface defects was affecting the clean regions we consider for our analysis, we would expect some anisotropy in the bias dependence of the CDW components (e.g. the stripes observed in [11]). This is not consistent with the perfectly homogeneous bias dependence of the three CDW component amplitudes depicted in Suppl. Fig. 6. All these experimental facts point to the same conclusion, that our multiband analysis is neither affected by proximity to the visible defects shown in Suppl. Fig. 5 nor by invisible subsurface defects.

VI. THE CDW IMAGING AMPLITUDE

In Suppl. Fig. 6a we show a map of the amplitude $a_1(\mathbf{r})$ obtained by fitting the CDW modulation along one of the three directions in Fig. 2a. The histogram of this amplitude map has a well defined peak, which we fit with a Gaussian to extract an amplitude $a_1(V_b)$ representative for the entire field-of-view for each imaging bias V_b. We repeat the same procedure for the other two directions to extract $a_2(V_b)$ and $a_3(V_b)$. All three a_i, (i=1,2,3), have a very similar bias dependence as seen in Suppl. Fig. 6b, prompting us to use their average at each bias in Fig 3b of the main text.

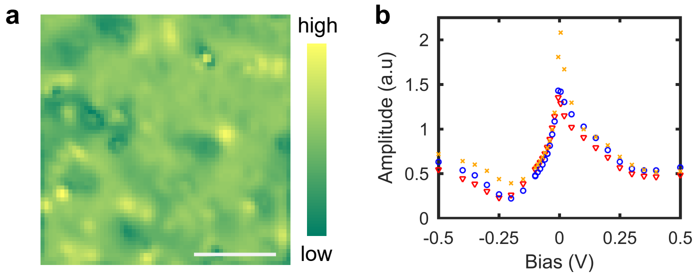

Suppl. Fig. 6. **The CDW imaging amplitude.** **a** Map of the local amplitude $a_1(\mathbf{r})$ of the CDW along one of the principle directions extracted from the STM image in Fig. 2b. Scalebar: 10 nm. **b** Bias dependence of the representative amplitudes a_i, (i=1,2,3) of each of the three one directional CDWs forming the CDW in Fig. 2.

VII. SPATIAL AND ENERGY DEPENDENCE OF THE LDOS IN A 1D CDW

In the following, we derive an expression of the energy and spatial dependence of the local density of states (DOS) in a 1D CDW. A similar derivation is presented in the supplementary material of ref. [12]. We start with the expressions from Grüner's book [13]:

$$\rho(x,k) = U_k^2 + V_k^2 - 2U_kV_k\cos(2k_Fx+\varphi), \tag{1}$$

where $U_k^2 = \frac{1}{2}\left(1-\frac{\epsilon_k}{E_k}\right)$ and $V_k^2 = \frac{1}{2}\left(1+\frac{\epsilon_k}{E_k}\right)$. $\epsilon_k = \hbar v_F(k-k_F)$ is the normal state dispersion linearised around the Fermi-energy and $E_k = \mathrm{sgn}(\epsilon_k)\sqrt{\epsilon_k^2+\Delta^2}$ is the dispersion in the CDW state, where Δ is the CDW energy gap. All energies are measured from the Fermi-energy. Inserting U_k and V_k into Supplementary Eq. 1 we get:

$$\begin{aligned}\rho(x,k) =& 1-\sqrt{1-\frac{\epsilon_k^2}{E_k^2}}\cos(2k_Fx+\varphi) = \\ =& 1-\sqrt{\frac{E_k^2-\epsilon_k^2}{E_k^2}}\cos(2k_Fx+\varphi) = \\ =& 1-\frac{\Delta}{E_k}\cos(2k_Fx+\varphi).\end{aligned} \tag{2}$$

We get the energy dependent local DOS from the following condition for the total number of states: $N = \int \rho(x,E)dE = \sum_k \rho(x,k) = \int_k \frac{L}{\pi}\rho(x,k)dk$ which yields:

$$
\begin{aligned}
\rho(x,E)dE =&\rho(x,k)\frac{L}{\pi}dk \\
\rho(x,E) =&\rho(x,k)\frac{L}{\pi}\frac{dk}{dE} = \rho(x,k)\frac{L}{\pi}\left(\frac{dE}{dk}\right)^{-1} \\
=&\rho(x,k)\frac{L}{\pi}\left(\mathrm{sgn}(\epsilon_k)\frac{\epsilon_k\hbar v_F}{\sqrt{\epsilon_k^2+\Delta^2}}\right)^{-1}
\end{aligned} \tag{3}
$$

Finally, by omitting the index for k:

$$
\begin{aligned}
\rho(x,E) =&\mathrm{sgn}(E)\frac{1}{\hbar v_F}\frac{L}{\pi}\left(1-\frac{\Delta}{E}\cos(2k_Fx+\varphi)\right)\left(\frac{\epsilon_k}{\sqrt{\epsilon_k^2+\Delta^2}}\right)^{-1} = \\
=&\left\{\text{using that } \epsilon_k^2 = E^2-\Delta^2 \text{ and that DOS at } \epsilon_F \text{ is } \mathcal{N}(E_F) = \frac{L}{\pi}\frac{1}{\hbar v_F}\right\} = \\
=&\mathrm{sgn}(E)\mathcal{N}(E_F)\left(1-\frac{\Delta}{E}\cos(2k_Fx+\varphi)\right)\left(\frac{E}{\sqrt{E^2-\Delta^2}}\right)
\end{aligned} \tag{4}
$$

VIII. CALCULATION OF THE CONSTANT CURRENT TOPOGRAPHY

We calculate 1D constant current topographic traces $z_0(x,V)$ by determining the tip-sample distance d at a given bias V and lateral position x that satisfies the constant current condition:

$$
I_{setpoint} - |I(d=z_0,x,V)| = 0, \tag{5}
$$

where $I_{setpoint} > 0$ is the set-point tunnelling current and $I(d,x,V)$ is the tunnelling current at a given tip-sample distance, lateral position and bias. To calculate the tunnelling current, we use the Bardeen equation for a 1D barrier in the limit of zero temperature [14]. First, we assume that the tip DOS does not depend on energy: $\rho_{tip}(E) = \rho_{tip}$. With this approximation, in the limit $T \to 0$, the tunnelling current is given by

$$
I(d,x,V) = \frac{4\pi e}{\hbar}\rho_{tip}\int_0^{eV}\rho_{sample}(x,E)\left|M(d,E,V)\right|^2 dE, \tag{6}
$$

where $M(d, E, V)$ is the tunnelling matrix element and $\rho_{sample}(x, E)$ is the local DOS of the sample. For the matrix element we use the result obtained for the 1D barrier [14]:

$$M(d, E, V) = C_M \frac{\hbar^2}{m} \kappa e^{-\kappa d}, \tag{7}$$

where C_M is a constant and κ is determined by the average work function of the tip and sample ($\Phi_0 = (\Phi_{tip} + \Phi_{sample})/2$) as

$$\kappa(V, E) = \sqrt{\frac{2m}{\hbar^2}\left(\Phi_0 + \frac{eV}{2} - E\right)} \tag{8}$$

Grouping all the constants in C_I, the tunnelling current reads

$$I(d, x, V) = C_I \int_0^{eV} \rho_{sample}(x, E) \kappa^2(V, E) e^{-2\kappa(V,E)d} dE. \tag{9}$$

Note that the above formalism is valid only for finite V. At $V = 0$ there is no net tunnelling current and the measurement cannot be performed. Formally, in the $V \to 0$ limit, $I \to 0$ as the integration range goes to zero. To maintain a finite current, the tip sample distance must be reduced $d \to -\infty$, meaning that the tip crashes into the sample. Technically speaking, it is not possible to record constant current images at zero bias as there is no tunnelling current for any positive tip-sample distance. In the calculations we avoid this situation by never evaluating the current at strictly zero bias.

We model the density of states of the sample $\rho_{sample}(x, E)$ by the sum of three terms: $\rho_{sample}(x, E) = \rho_0 + \rho_1(x, E) + \rho_2(x, E)$. ρ_0 is a constant background while ρ_1 and ρ_2 are the DOS variations due to the two CMs:

$$\rho_1(x, E) = \Re\left(\mathrm{sgn}(E - \varepsilon_1)\left(1 - \frac{\Delta_1}{(E + i\Gamma - \varepsilon_1)}\cos(k_{CDW}x)\right)\frac{(E + i\Gamma - \varepsilon_1)}{\sqrt{(E + i\Gamma - \varepsilon_1)^2 - \Delta_1^2}}\right) \tag{10}$$

and

$$\rho_2(x,E) = \Re\left(\mathrm{sgn}(E-\varepsilon_2)\left(1-\frac{\Delta_2}{(E+i\Gamma-\varepsilon_2)}\cos(k_{CDW}x-\varphi)\right)\frac{(E+i\Gamma-\varepsilon_2)}{\sqrt{(E+i\Gamma-\varepsilon_2)^2-\Delta_2^2}}\right), \quad (11)$$

where $\Delta_{1,2}$ are the size of the gaps, $\varepsilon_{1,2}$ are the energies where the gaps are centred (gap midpoint) with respect to E_F, $k_{CDW} = 2\pi/\lambda_{CDW}$ is the CDW ordering vector, λ_{CDW} the wavelenght and φ a real-space phase difference between the two CMs. We used $\Gamma = 0.005$ eV for all the calculations presented in this work.

In order to calculate the constant current topographic traces, we solve numerically Supplementary Eq. 5 where we use Supplementary Eq. 9 for the tunnelling current and $\Phi_0 = 5.2$ eV for the average tip-sample work function (W and $NbSe_2$). To simulate a realistic situation we set C_I and ρ_0 such that the average tip-sample distance at a given bias (away from zero) is in the 5-8 Å range when the tunnelling current setpoint is 100 pA. The phase and amplitude at a given bias is simply determined from the position of the maximum and from the difference of the maximum and minimum value in the calculated constant current topographic traces.

IX. BIAS DEPENDENCE OF THE CDW AMPLITUDE IN THE DIFFERENT MODELS

In Suppl. Fig. 7, we present the bias dependence of the CDW amplitude calculated using the above method for the three models discussed in Fig. 4 of the main text.

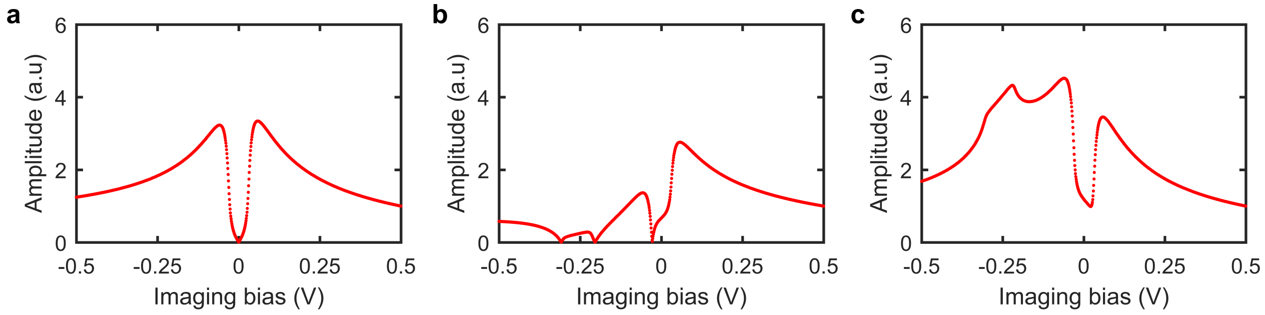

Suppl. Fig. 7. **Bias dependence of the amplitude of the CDW signal of the STM topography in the different 1D situation discussed in Fig 4.** **a** A single CM with a gap at the Fermi level. **b** Two CMs with the two gaps centred at different energies, but without any real-space phase difference between the CMs. **c** Same as **b**, but with a 120° ($2\pi/3$) phase difference between the CMs. For clarity, all the curves are normalized to their value at $V_b = 0.5$ V.

X. OPTIMIZED PARAMETERS OF THE TWO-GAP MODEL

To compare the two-gap model with our experimental data, we performed a visual optimization of the main parameters of the model: the size of the two gaps ($\Delta_{1,2}$) and their midgap position ($\varepsilon_{1,2}$). The model shows an excellent qualitative agreement with the experimental data (Fig. 3) for the following parameter ranges: $\Delta_1 \in [8, 16]$ meV, $\Delta_2 \in [45, 75]$ meV, $\varepsilon_1 \in [-14, -2]$ meV and $\varepsilon_2 \in [-100, -65]$ meV, where $\varepsilon_{1,2}$ are measured from E_F. With the parameters in these ranges the model reproduces the overall line-shape of the bias dependence of both the amplitude and the phase, and all the main features of the experimental data as discussed in the main text. In Figs. 3c and d we plot the bias dependence of the phase and amplitude obtained in the model using the parameters at the middle of the above ranges: $\Delta_1 = 12$ meV, $\Delta_2 = 60$ meV, $\varepsilon_1 = -8$ meV and $\varepsilon_2 = -82.5$ meV.

XI. PHASE AND AMPLITUDE IN THE THREE-GAP MODEL

We have also calculated the bias dependence of the phase and amplitude in a three-gap model by considering one more gap and a concomitant CM. The third CM we set in-phase with the second, i.e 120° out-of-phase to the first. In Suppl. Fig. 8 we use the same size and position of the gap ($\Delta_1 = 12$ meV and $\varepsilon_1 = -8$ meV) for the first CM as in the optimized two-gap model, and we split the second gap of the optimized two-gap model into two for the second and third CM of the three-gap model: $\Delta_2 = 30$ meV, and $\varepsilon_2 = -43$ meV, $\Delta_3 = 30$ meV, and $\varepsilon_3 = -123$ meV. The obtained bias dependence of the phase and amplitude of the CDW signal (Suppl. Figs. 8c and d) shows similarly good qualitative agreement with the experimental data (Suppl. Figs. 8a and b) as in the two-gap model (Fig. 3)

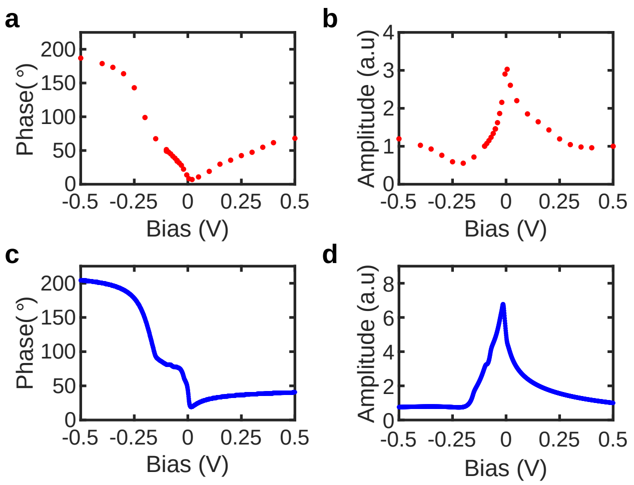

Suppl. Fig. 8. **Phase and amplitude in the three-gap model.** **a**, **b** Bias dependence of the phase and amplitude of the CDW signal in experiments (the same as in Fig. 3) and **c**, **d** in a three-gap model model. The data in **b** and **d** are normalized to their $V_b = 0.5$ V value.

[1] Á. Pásztor, A. Scarfato, M. Spera, C. Barreteau, E. Giannini, and C. Renner, Holographic imaging of the complex charge density wave order parameter, Physical Review Research **1**, 033114 (2019).

[2] W. L. McMillan, Theory of discommensurations and the commensurate-incommensurate charge-density-wave phase transition, Phys. Rev. B **14**, 1496 (1976).

[3] G. Gye, E. Oh, and H. W. Yeom, Topological landscape of competing charge density waves in 2H-$NbSe_2$, Physical Review Letters **122**, 016403 (2019).

[4] B. Guster, C. Rubio-Verdú, R. Robles, J. Zaldívar, P. Dreher, M. Pruneda, J. . Silva-Guillén, D.-J. Choi, J. I. Pascual, M. M. Ugeda, P. Ordejón, and E. Canadell, Coexistence of elastic modulations in the charge density wave state of 2H-NbSe2, Nano Letters **19**, 3027 (2019).

[5] H. Fukuyama, Pinning in Peierls-Fröhlich state and conductivity, J. Phys. Soc. Jpn. **41**, 513 (1976).

[6] H. Fukuyama and P. A. Lee, Dynamics of the charge-density wave. I. Impurity pinning in a single chain, Phys. Rev. B **17**, 535 (1978).

[7] B. Hildebrand, T. Jaouen, C. Didiot, E. Razzoli, G. Monney, M. L. Mottas, A. Ubaldini, H. Berger, C. Barreteau, H. Beck, D. R. Bowler, and P. Aebi, Short-range phase coherence and origin of the 1T-$TiSe_2$ charge density wave, Phys. Rev. B **93**, 125140 (2016).

[8] A. M. Novello, M. Spera, A. Scarfato, A. Ubaldini, E. Giannini, D. R. Bowler, and C. Renner, Stripe and short range order in the charge density wave of 1T-Cu_xTiSe_2, Phys. Rev. Lett. **118**, 017002 (2017).

[9] U. Chatterjee, J. Zhao, M. Iavarone, R. Di Capua, J. P. Castellan, G. Karapetrov, C. D. Malliakas, M. G. Kanatzidis, H. Claus, J. P. C. Ruff, F. Weber, J. van Wezel, J. C. Campuzano, R. Osborn, M. Randeria, N. Trivedi, M. R. Norman, and S. Rosenkranz, Emergence of coherence in the charge-density wave state of 2H-$NbSe_2$, Nature Communications **6**, 6313 (2015).

[10] W. Jolie, T. Knispel, N. Ehlen, K. Nikonov, C. Busse, A. Grüneis, and T. Michely, Charge density wave phase of VSe_2 revisited, Physical Review B **99**, 115417 (2019).

[11] A. Soumyanarayanan, M. M. Yee, Y. He, J. van Wezel, D. J. Rahn, K. Rossnagel, E. W. Hudson, M. R. Norman, and J. E. Hoffman, Quantum phase transition from triangular to

stripe charge order in $NbSe_2$, Proc. Natl. Acad. Sci. **110**, 1623 (2013).

[12] J. Dai, E. Calleja, J. Alldredge, X. Zhu, L. Li, W. Lu, Y. Sun, T. Wolf, H. Berger, and K. McElroy, Microscopic evidence for strong periodic lattice distortion in two-dimensional charge-density wave systems, Physical Review B **89**, 165140 (2014).

[13] G. Grüner, *Density waves in solids* (Westview Press, 2000).

[14] B. Voigtländer, *Scanning probe microscopy: Atomic force microscopy and scanning tunneling microscopy* (Springer, 2015).
