# Supplementary material for: Multiband charge density wave exposed in a transition metal dichalcogenide
Source: Nat Commun. 2021 Oct 15;12:6037. doi: 10.1038/s41467-021-25780-4 (PMC8519912; doi:10.1038/s41467-021-25780-4)
Supplement: Supplementary file 3 — Editorial Assessment Report [file 41467_2021_25780_MOESM3_ESM.pdf]

Editorial Assessment Report

Dear Professor Renner,

Thank you again for choosing to submit your manuscript using the Guided Open Access pilot at the Nature Portfolio. As part of this process, our editorial team has considered your paper for three of our journals with strong interest in publishing in your field: *Nature Physics*, *Nature Communications*, and *Communications Physics*.

Your manuscript has now been reviewed by three experts in two-dimensional materials, CDWs, scanning tunneling microscopy/spectroscopy, and the relevant theory. We have included their comments in this Editorial Assessment Report. As part of the Guided Open Access pilot, editors from all three journals have discussed the reviewer reports and the manuscript's suitability for our journals. After careful evaluation, our editorial recommendation is to revise the manuscript with additional theoretical work and submit back through the Guided Open Access submission portal for further consideration at Nature Communications using the link provided in the 'Next Steps' section below.

To unpack this a little more, for publication in *Nature Physics*, more experimental work is required, as the reviewers have expressed concerns about the extent to which sample quality may be impacting the CDW formation. We can also consider this work further for *Nature Communications* if you include more convincing theoretical analysis. Lastly, we will be happy to publish this work in *Communications Physics* with only minor changes and modified claims. We have provided more detail on each of these options below, but as I say, our editorial guidance is to aim for the second of them.

Please note that the Editorial Assessment Report is a standalone document that contains an editorial evaluation, recommendation and portable peer advice to help you navigate and interpret the reviewers' reports. It also provides guidance for adhering to best practice with regard to transparency and reproducibility, for example on the issue of sharing data. We have also included information about data accessibility and reproducibility, which we hope you find useful.

Yours sincerely,
David
--
Dr David Abergel
Senior Editor, *Nature Physics*
On behalf of the Guided OA editorial team

nature portfolio

GUIDED OPEN ACCESS

nature physics | nature communications | communications physics

Contents of this Report

- **Manuscript overview**: details about your manuscript and the editorial team.
- **Manuscript assessment**: personalised recommendation from the editors.
- **About the editorial process**: an overview of the Guided Open Access process.
- **Annotated reviewer comments**: the referee reports with comments from the editors.
- **Open research evaluation**: advice for adhering to best reproducibility practices.

Manuscript overview

Manuscript number	Submission date	Decision date
GUIDEDOA-21-00008	12 January 2021	12 March 2021

**Title**	Multiband charge density wave exposed in a transition metal dichalcogenide	**Corresponding author**	Christoph Renner ORCID: 0000-0001-9882-681X
**Preprint information**	A preprint version of this manuscript is available on arXiv: https://arxiv.org/abs/2102.00025	**Peer review type**	Single-blind
**Editorial Assessment Team**	**Primary editor: David Abergel**<br>**Home Journal:** *Nature Physics*, ORCID: 0000-0002-6166-181X<br>**Editorial team members:**<br>Wei Fan, *Nature Communications*, ORCID: 0000-0003-3629-6053<br>Saleem Denholme, *Communications Physics*, ORCID: 0000-0001-6017-0819		
**About your primary editor**	Before joining *Nature Physics* in 2017, David carried out theoretical research on graphene and other two-dimensional crystals, and quantum topological materials. He completed a Ph. D at Lancaster University (UK) in 2007, and then did post-doctoral work at the University of Manitoba (Canada) and the University of Maryland (USA) before undertaking an Assistant Professorship at Nordita, the Nordic Institute for Theoretical Physics in Stockholm, Sweden. David is based in our London office.		

Manuscript assessment and recommendation

**Editor's summary of the manuscript and overall assessment**	The main claim in this manuscript is the observation of a multi-band charge-density wave in $NbSe_2$, meaning that a gap opens on two bands simultaneously. This observation is made using the author's previously-discussed STM technique that analyses the change in the topography as a function of tip-sample bias.

Editorial assessment overview

Nature Physics	**Major revisions needed**	More experimental work needed.
Nature Communications	**Some additional data needed, but generally minor revisions**	More theoretical work needed.
Communications Physics	**Minor revisions needed**	Publish with weakened claims.

**Editor's recommendation**	***Option 1*: Revise for consideration at Nature Physics**<br>In order to move towards publication in Nature Physics, we would need to see the experiments repeated using cleaner samples to satisfy the request of Reviewer #1.<br><br>***Option 2*: Revise for consideration at Nature Communications**<br>In order to move towards publication in Nature Communications, a more detailed theoretical analysis is required to satisfy the requests of Reviewers #1 and #2.<br><br>***Option 3*: Revise for consideration at Communications Physics**<br>This work can be published in Communications Physics with minor revisions and clarifications.

**Next steps**	If you would like to follow our recommendation, when you are ready you can upload the revised manuscript, along with your point-by-point response to the reviewer's reports and editorial advice **here**.

About the editorial process

By selecting the Nature Portfolio Guided Open Access option, your manuscript was assessed for suitability in three of our titles that provide venues for publication of high-quality work across the spectrum of physics research: *Nature Physics, Nature Communications*, and *Communications Physics*. For more information about Guided Open Access, please see here.

Collaborative editorial assessment

Your editorial team discussed the manuscript to determine its suitability for the Nature Portfolio Guided OA pilot. Our assessment of your manuscript takes into account several factors, including whether the work meets the **technical standard** of the Nature Portfolio and whether the findings are of **immediate significance** to the readership of at least one of the participating journals in the Nature Portfolio Guided Open Access physics cluster.

Peer review

Experts were asked to evaluate the following aspects of your manuscript:

Novelty in comparison to prior publications;

Likely audience of researchers in terms of broad fields of study and size;

Potential impact of the study on the immediate or wider research field;

Evidence for the claims and whether additional experiments or analyses could feasibly strengthen the evidence;

Methodological detail and whether the manuscript is reproducible as written;

Appropriateness of the literature review.

Editorial evaluation of reviews

Your editorial team discussed the potential suitability of your manuscript for each of the participating journals. They then discussed the revisions necessary in order for the work to be published, keeping each journal's specific editorial criteria in mind.

Journals in the Nature portfolio will support authors wishing to transfer their reviews and (where reviewers agree) the reviewers' identities to journals outside of Springer Nature. For any questions about review portability, please contact our editorial office: guidedoa@nature.com

Annotated Reviewer Reports

The editor has included some additional comments (black text) on the specific points raised by the reviewers (blue text) below. However, please note that all points should be addressed in a revision, even if the editor has not specifically commented on them.

Reviewer #1

Report received: 10 February 2021

**Reviewer #1**	This reviewer has not chosen to waive anonymity. The reviewer's identity can only be shared with representatives of an established journal editorial office.
**Reviewer #1 expertise** *Summarised by the editor*	Scanning tunneling microscopy/spectroscopy on 2D materials
**Editor's comments about this review**	The overall message that we take from this review is that the claim of multiple CDWs is not fully substantiated because the samples are not sufficiently clean and the theoretical treatment may not be capturing the relevant physics.

Reviewer #1 comments

**Overview**	The authors utilize scanning tunneling microscopy, in combination with theoretical approaches to understand the charge density waves in the prototypical system of $NbSe_2$. In contrast to the established opinions of this long studied system, they claim that the origin of the ordering stems from the formation of two CDWs originating from two bands. They substantiate this claim, by spatially dependent imaging of the CDW and relating this to a two-gap model. While the claim is certainly impactful, should it be substantiated, I do not believe that the evidence in the manuscript proves the main claims of this paper. Likewise, as neither the material, nor the methods used to study this paper do not present a new state of the art, I cannot recommend this for publication in Nature Physics.

Specific comments

#	Reviewer comment	Editorial comment
1	From the experimental standpoint, my main issue with the paper has to do with the quality of the material. The images shown in Fig. 2 show a large defect density in the crystal, with the presence of both surface and many subsurface defects. It has been shown that defects in $NbSe_2$ can lead to a change in the observed CDW (e.g. see	Given that defects are well known to have an impact on the CDW, this seems like a substantial and important comment.

	PNAS 2013 110 (5) 1623-1627). In order to make the claims the authors are making, it is vital to study a cleaner and more defect free crystal, in order to exclude that modifications of the CDW stem from modulations of the electronic structure stemming from such defects, e.g. electronic disorder. Considering this materials has been studied for decades, and in better quality, understanding this point is essential if the authors which to claim their experiments refute the previous understandings.	
**2**	Moreover, theoretically, I found myself questioning if the theoretical treatment is accurate enough to treat the problem. In the case, that one is interested in looking at multi-band CDW formation, then I would expect it is necessary to accurately describe long-range correlations in this system. The latter is extremely challenging theoretically, requiring advanced methods (e.g. dual boson, etc.), which may lead to small but important corrections to the electronic structure (e.g. Fig. 5). The authors make no appeal as to how correlations are accurately described, and why more sophisticated treatments can be neglected (contrary to literature). I found next to no description of the theoretical methods in the paper or supplement to help substantiate this. While I do not insist that such computations need to be performed, as this is unrealistic, the authors should properly address their approach and why or why not these other approaches are not relevant, or what they expect if such computations were to be performed.	The referee does not ask for explicit computations of the role of the long-range interactions, but we would like to see more discussion of this issue. See also the comments of Reviewer #2 below.
**3**	If the authors are able to study a clean material and substantiate the above points concerning the theory, this may be reconsidered for Nature Physics. Otherwise, I believe this paper presents more doubt that makes a breakthrough, and I would otherwise only consider this for Communication physics, if the claims are relaxed and the points properly addressed.	

**Reviewer #2**<br>*Report received: 4 March 2021*	
**Reviewer #2**	This reviewer has not chosen to waive anonymity. The reviewer's identity can only be shared with representatives of an established journal editorial office.

**Reviewer #2 expertise** *Summarised by the editor*	Computational descriptions of 2D materials, including TMDs. Also has worked on CDWs.
**Editor's comments about this review**	This report is a little brief, but positive about the overall impact of multiple CDWs, if this is proven with sufficient rigour. The clear message is that more convincing theoretical work is needed to make the claim of multiple CDWs more compelling.
**Reviewer #2 comments**	
**Overview**	The manuscript by Renner et al. reports an experiment and theory collaboration regarding on multiple charge density wave (CDW) on surface of 2H-NbSe2. The work is largely based on their previous works on the same material. In this work, they developed a mapping method from two dimensional charge orders (three-fold CDWs) to one-dimensional one and then extract phase and amplitude of the CDW. From phenomenological fitting, they conclude that observed scanning tunneling spectroscopic charge modulations can be explained by including at least two different charge orders, one at the Fermi energy and the other at the different energy.<br><br>As the authors stressed in the manuscript, I also agree that their study on the multiple CDWs with different energetic positions is first among many. Usually, as the authors mentioned, the multiple CDW orders have been discussed in the context of symmetry or multiple Q (nesting momentum) order. Hence, I would like to value the new claim on the multiband CDW very highly. However, the work, specially, analysis is quite phenomenological and the persuasive theoretical confidence compared with multiband superconductivity is lacking. So, in the process of the Guided Open Access program, I recommend the work be suitable for publication in Nature Communication or others, not in Nature Physics.

Specific comments

#	Reviewer comment	Editorial comment
1	This system is quite simple and electron-phonon interaction or other physical quantities related with CDWs can be computed in the level of atomistic calculations. If the CDW orders are indeed multiband origins, the author can compute and can show the gap openings at some specific electronic energy bands as well as phononic dispersion from first-principles. Since the energy dependent self-consistent formulation for electron-phonon coupling similar to Eliashberg formalism for the multiband superconductivity can compute in principle, the conclusive theoretical evidence for multiband CDW can be	Similar to Reviewer #1, this reviewer would like a more rigorous theoretical treatment.

	obtain with a similar approach.	
**2**	Actually, I found no evidence or theoretical reasons why another CDW order occurs at -0.25 eV since the authors provide only phenomenological fitting data. So, later, it would be very persuasive and decisive work if the authors can show these data. For the moment, the concept of multiband CDWs is new and interesting but solid support for the claim is lacking.	

Reviewer #3
Report received: 8 March 2021

**Reviewer #3**	This reviewer has not chosen to waive anonymity. The reviewer's identity can only be shared with representatives of an established journal editorial office.
**Reviewer #3 expertise** *Summarised by the editor*	Scanning tunneling microscopy/spectroscopy on strongly-correlated materials.
**Editor's comments about this review**	This report is clearly positive, with only minor suggestions for improvements. This is what motivates us to offer the option for further consideration at Nature Physics if the phenomenology is reproduced in cleaner samples.
**Reviewer #3 comments**	
**Overview**	The manuscript by Pasztor et al. reports on real-space evidence for the multiband nature of 2D charge density wave (CDW) order in an archetypal dichalcogenide material 2H-NbSe2. By systematic model analysis of atomic-scale topography data taken with crygenic scanning tunneling microscopy (STM) at various tip-sample bias voltages, particularly how the CDW modulations in the form of topographic STM image contrast depend on bias voltage, this work presents direct and detailed agreement of the STM topography data with a well-grounded CDW model based on three CDWs that are commensurate with the atomic lattice and on two de-phased charge modulations (CM) arising from two principal energy bands. The analytical methodology used is original, in that it extends well-established CDW formalism to multiple energy bands. The key results reported are of both breakthrough and general significance, in demonstrating a novel technique to extract real-space phase information about spatially-modulated electronic orders. Furthermore, the revelation

	of CDW energy gaps well away from the Fermi level represents crucial evidence for strong electron correlations in the formation of CDW order in 2H-NbSe2.

Specific comments

#	Reviewer comment	Editorial comment
1	In my opinion, this manuscript warrants appearance in Nature Physics, because its results shed new light on a widely-studied though still-enigmatic CDW material, and also because the novel methodology it demonstrates can be generally applied to a variety of complex 2D materials as well as nanoengineered heterostructures (epitaxial, van der Waals or Moire pattern).	We are taking this comment about the generality of the method into account in our decision.
2	This work is convincing, in terms of its phenomenological clarity that connects the STM topography data to the multiband CDW model. Of particular merit is the transparent choice of model parameters, which are either constrained by the specific lattice structure of 2H-NbSe2 (via the tight-binding band structure) or based on general physical considerations (especially that the phase shift between charge modulations of the two bands is determined by the minimization of Coulomb-repulsion as well as lattice-commensuration energies).	This somewhat goes against the recommendations of the other two reviewers, but we are taking this point of view on board.
3	Experimentally, was any temperature dependence observed in the STM image contrast dependence on bias, both below and even above the CDW ordering temperature of 2H-NbSe2? Theoretically, would one expect temperature to affect the relative strengths between the Coulomb-repulsion and lattice-commensuration energies?	It would be good to revise the manuscript in response to points 3-7 here regardless of which resubmission option you choose.
4	What justifies neglecting the "pancake" Fermi surface (FS) sheet (centered near the Gamma point) in the multiband CDW model analysis? Was it simply because of its very small phase space relative to the other FS sheets? Or perhaps the 3D geometry of the pancake FS sheet, in contrast to the 2D tubular FS sheets, renders it less conducive to the in-plane CDW order?	
5	What does the appearance of energy gaps away from the Fermi level imply about the role that strong electron correlations play in the formation of CDWs in 2H-NbSe2, since band structure calculations by Johannes, Mazin and Howells (Ref. 11) had shown that conventional	

	mechanism based predominantly on Fermi surface nesting does not adequately explain the observed CDW order.	
**6**	For clarity in the caption of Figure 4, specify the color coding of the middle column of panels (b, e, h) in order to disambiguate it from that of the right column of panels (c, f, i).	
**7**	In Line 243, perhaps replace the word "backing" by "supporting".	

Open Research Evaluation	
**Data Availability**	
**Recommended data deposition**	The Nature Portfolio journals strongly support public availability of data associated with a manuscript in a persistent repository where they can be freely and enduringly accessed or as a supplementary data file when no appropriate repository is available. For more information, please refer to our page on reporting standards and availability of data, materials, code and protocols.<br><br>We recommend that all relevant raw or processed experimental data in your study be made available at the point of publication on a public repository such as Figshare or Zenodo, so that they can be referred with unique digital object identifiers.<br><br>In addition, we strongly encourage you to upload the Source Data (that is, the specific data shown in the figures in the main text and Extended Data) along with your resubmission.<br><br>If you need help complying with this policy, or need help depositing and curating your research data (including raw and processed data, text, video, audio and images) you should consider:<br>● Contacting Springer Nature's Research Data Helpdesk for advice,<br>● finding a suitable data repository for your data,<br>● Or uploading your data to Springer Nature's Research Data Support service.<br><br>Please note there are fees for using Springer Nature's Research Data Support service. You may also find more information on our policy page.
**Data Availability Statement**	All papers published by the Nature Portfolio must include a Data Availability Statement (DAS).<br><br>A DAS should include, at a minimum, a statement confirming that all relevant data are available from the authors, and/or

	are included with the manuscript (e.g. as source data), listing which data are included (e.g. by figure panels and data types) and mentioning any restrictions on availability. If a dataset generated or analysed during the study is publicly available and has a Digital Object Identifier (DOI) as its unique identifier, we strongly encourage including this in the Reference list and citing the dataset in the Methods.<br><br>In the present case, we suggest that you amend your DAS as follows:<br><br>“Source data are available for this study. All other data that support the plots within this paper and other findings of this study are available from the corresponding author upon reasonable request”.<br><br>However, please feel free to amend this statement, especially if you are able to share more of your data.
**Source data**	The following figure panels should be accompanied by the underlying source data: **3, 4b,c,e,f,h,i, 5**. In addition, we can also accommodate the data shown in the 2D plots in Figs 2 and 4 if you wish to share it.
**Data citation**	If a dataset generated or analysed during the study is publicly available and has a DOI as its unique identifier, we strongly encourage including this in the Reference list and citing the dataset in the Methods.<br><br>Citing and referencing data in publications supports reproducible research, by increasing the transparency and provenance tracking of data generated or analysed during research. Citing data formally in reference lists also helps facilitate the tracking of data reuse and may help assign credit for individuals’ contributions to research. A number of Springer Nature imprints are signatories of the Joint Declaration on Data Citation Principles, which stress the importance of data resources in scientific communication.
**Code Availability**	
**Code Availability**	As for data, the Nature Portfolio journals strongly support

	public availability of custom code associated with a manuscript in a persistent repository where they can be freely and enduringly accessed, where appropriate.
**Code Citation**	In addition to making the custom code available, we suggest that you ensure that the version of the code/software described in the paper is deposited in a DOI-minting repository such as Zenodo and that this DOI is also cited in the main Reference list. See here for more details.
**Research ethics**	
**Competing Interests**	In the interests of transparency and to help readers form their own judgements of potential bias, Nature Portfolio journals require authors to declare any competing financial and/or non-financial interests in relation to the work described.<br><br>Please provide a 'Competing interests' statement using one of the following standard sentences:<br><br>• The authors declare the following competing interests: [specify competing interests]<br><br>• The authors declare no competing interests.<br><br>See our competing interests policy for further information: https://www.nature.com/nature-research/editorial-policies/competing-interests
**Methods assessment and reproducibility**	
**Methods Descriptions**	The Methods must contain sufficient detail such that the work could be replicated. It is preferable that all key methods be included in the main manuscript, rather than in the Supplementary Information.<br><br>The methods section can be up to 3,000 words in length, they can contain references that do not count towards the reference limit in the main paper, and will be fully indexed. You should feel free, and we in fact encourage you, to incorporate any part of your Supplementary Information that you feel is important for the rest of the paper within this section.

	The Methods section should be written as concisely as possible but should contain all elements necessary to allow interpretation and reproduction of the results (please note, however, that the methods section cannot contain any figures or tables at present).<br>If there are additional references in the Methods section, their numbering should continue from the last reference in the main paper, and the list should follow the Methods section.
**Error bars and statistics**	Error bars should be displayed wherever possible and must be clearly defined in the caption for each figure.
